# A Systematic Review and Meta-Analysis on Depression and Associated Factors among Adult HIV/AIDS-Positive Patients Attending ART Clinics of Ethiopia: 2021

**DOI:** 10.1155/2021/8545934

**Published:** 2021-10-20

**Authors:** Bitew Tefera Zewudie, Shegaw Geze, Yibeltal Mesfin, Muche Argaw, Haimanot Abebe, Zebene Mekonnen, Shegaw Tesfa, Bogale Chekole, Betelhem Tadesse, Agere Aynalem, Tadele Lankrew

**Affiliations:** ^1^Department of Nursing, College of Medicine and Health Science, Wolkite University, Wolkite, Ethiopia; ^2^Department of Midwifery, College of Medicine and Health Science, Wolkite University, Wolkite, Ethiopia; ^3^Department of Nursing, College of Medicine and Health Science, Wolaita Sodo University, Wolaita, Ethiopia

## Abstract

**Introduction:**

Depression is the most common mental health problem in people living with the human immune virus. It ranges from 11% to 63% in low- and middle-income countries. Depression was high in people living with HIV/AIDS in developing countries, especially in the Ethiopian context. Even though depression has negative consequences on HIV-positive patients, the care given for depression in resource-limited countries like Ethiopia is below the standard in their HIV care programs.

**Method:**

International databases (Google Scholar, PubMed, Hinari, Embase, and Scopus) and Ethiopian university repository online have been covered in this review. Data were extracted using Microsoft Excel and analyzed by using the Stata version 14 software program. We detected the heterogeneity between studies using the *I*^2^ test. We checked publication bias using a funnel plot test.

**Results:**

The overall pooled depression prevalence among adult HIV/AIDS patients attending antiretroviral therapy in Ethiopia was 36.3% (95% CI: 28.4%, 44.2%) based on the random effect analysis. Adult HIV/AIDS patients having CD4count < 200(AOR = 5.1; 95% CI: 2.89, 8.99), widowed marital status (AOR = 3.7; 95% CI: 2.394, 5.789), medication nonadherence (AOR = 2.3; 95% CI: 1.63, 3.15), poor social support (2.986) (95% CI: 2.139, 4.169), perceived social stigma (2.938) (2.305, 3.743), opportunistic infections (3.010) (2.182, 4.151), and adverse drug reactions (4.013) (1.971, 8.167) were significantly associated with depression among adult HIV/AIDS patients on antiretroviral therapy, in Ethiopia. *Conclusion and Recommendation*. The pooled depression prevalence among adult HIV/AIDS patients attending antiretroviral therapy in Ethiopia was higher than the general population and is alarming for the government to take special consideration for HIV-positive patients. Depression assessment for all HIV-positive patients and integrating with mental health should be incorporated to ensure early detection, prevention, and treatment. Community-based and longitudinal study designs mainly focusing on the incidence and determinants of depression among adult HIV/AIDS patients should be done in the future.

## 1. Introduction

Depression is the most common mental health problem in people living with the human immune virus (PLHIV). In low- and middle-income countries, it ranges from 11% to 63% [1, 2]. Human immune deficiency virus/acquired immune deficiency syndrome (HIV/AIDS) is a global problem, especially in sub-Saharan Africa countries. Ethiopia is one of the sub-Saharan countries that is affected by the problem [3]. Globally, around 37.9 million people were living with HIV/AIDS, and more than 35 million deaths have occurred since its emergence. In sub-Saharan Africa, HIV/AIDS prevalence was 4.2% and contributes 1/3 of the world prevalence, and Ethiopia is one of the sub-Saharan Africa countries accounting for 0.9% HIV/AIDS prevalence [4–6].

Research findings revealed that depression is nearly three times more common in HIV-positive patients than HIV-negative individuals [7]. According to mental health statistics report, depression is ranked third in the global burden of disease. However, by 2030, it will be the leading cause of disease burden [8]. Depression was high in people living with HIV/AIDS in developing countries, especially in the Ethiopian context. Studies conducted across different regional states of Ethiopia assessed depression prevalence among HIV-positive patients on ART. The depression prevalence among HIV/AIDS patients in Bahir Dar northwest Ethiopia was 46.8% [9], in eastern Ethiopia 45.8% [10], in Axum northern Ethiopia 14.6% [11], in Gimbi Hospital west Ethiopia 41.7% [12], and in Hawassa southern Ethiopia 48.6% [13]. The pooled depression prevalence among HIV/AIDS patients in sub-Saharan Africa was 31.2% and 38% in east Africa [14].

Treatment resistance, recurrence of opportunistic infections, increased demand for utilization of medical resources, increased morbidity and mortality, and, finally, poor health outcomes of HIV-positive patients were mainly due to the comorbidity of depression on HIV/AIDS [15]. Even if the Federal Ministry of Health (FMOH) of Ethiopia committed to addressing the needs of their people for effective, affordable, equitable, and sustainable mental health services, depression among PLWHIV still did not get adequate attention as basic HIV care service [15].

Nowadays, depression among patients living with HIV/AIDS imposes various socioeconomic and negative health impacts on the public. Therefore, it is crucial to have new strategies and countrywide planning to maintain the mental as well as physical health of these individuals [16]. So it is vital to determine the statistical magnitude and factors mainly associated with depression in patients living with HIV/AIDS at the country level. In spite of its negative consequences and high burden among HIV-positive patients, the standard of care for depression in resource-limited low-income countries like Ethiopia is poor in their HIV care programs [17–19]. Therefore, the objective of this review was to assess the national pooled depression prevalence and its associated factors among HIV/AIDS patients attending ART clinics in Ethiopia.

## 2. Methods and Materials

### 2.1. Study Design and Search Strategy

We searched studies of depression prevalence and associated factors among adult HIV/AIDS patients attending ART in Ethiopia. We included studies found in Google Scholar, African Journals Online, PubMed/MEDLINE, Hinari, Embase, Scopus, and Ethiopian university repository online. Studies published from 2015 to 2021 were included in this systematic review. Articles were searched using the MESH terms as “prevalence of depression AND associated factors OR adult HIV/AIDS patients and ART.” We strictly follow the Preferred Items for Systematic Review and Meta-Analysis (PRISMA) guideline and protocol to write this systematic review and meta-analysis.

### 2.2. Eligibility Criteria

#### 2.2.1. Inclusion Criteria

Eligible articles for this systematic review and meta-analysis were studies that assessed depression among adult HIV/AIDS patients attending ART clinics, conducted in Ethiopia, and studies that assessed factors associated with depression. We included all cross-sectional studies about depression prevalence and associated factors among adult HIV/AIDS patients attending ART clinics of Ethiopia and written in the English language in the review. Studies among adult (age ≥ 18 years) HIV-positive patients on ART follow-up were included. We searched for the presence of articles similar to our title in 10 regions of Ethiopia. However, we found articles only in five regions of Ethiopia which had similar characteristics to our review.

#### 2.2.2. Exclusion Criteria

Duplicated articles, articles unable to access the full text, and abstract were removed from the review. Moreover, articles published in the non-English language, interventional studies, and without a tool to screen depression were excluded from this review.

### 2.3. Data Extraction and Quality Assessment

Information such as first author name, sample size, regions of the study, study design, date of publication, tools used to assess the outcome variable, OR, and 95% CI were parameters used in the data extraction template. We used standardized Microsoft Excel spreadsheet data extraction form to extract relevant information from eligible articles. Data extraction from included articles was done independently by the authors (YM, BTZ, HA, ZM, BC, MA, and SG). Disagreements were resolved by discussion and common consensus with the rest of the authors. Articles that fulfilled the eligibility criteria were included in the final analysis after summarizing in the table.

#### 2.3.1. Outcome Variable

The first outcome of interest for this systematic review and meta-analysis was depression prevalence among adult HIV/AIDS patients attending ART. And the pooled depression prevalence among adult HIV/AIDS patients attending ART was computed. The second outcome variable is factors associated with depression among adult HIV/AIDS patients attending ART and was computed by using log odds ratio. Ten of the studies included in our review used PHQ-9 as a measuring tool to assess depression prevalence, whereas a study conducted in the Amhara region by Tareke et al. used HAD-D.

#### 2.3.2. Data Processing and Analysis

We used standardized Microsoft Excel spreadsheet data and Stata version 14 software for data extraction and analysis of extracted data, respectively. Random effect model meta-analysis was used to compute pooled depression prevalence. Because eleven studies were included in the final analysis and some of the studies used different scales, the unstandardized and standardized regression coefficients of the random effect analysis model were used. Publication bias was checked by funnel plot via visual assessment. Heterogeneity between studies was checked by using the Cochran *Q* statistic and *I*^2^ test. Subgroup analysis by region was computed to compare depression prevalence within regions of Ethiopia. Point prevalence was presented by forest plot format with 95% CI. And associated factors with depression were assessed by using AOR.

## 3. Result

### 3.1. Identification of Included Studies

**Figure 1:**
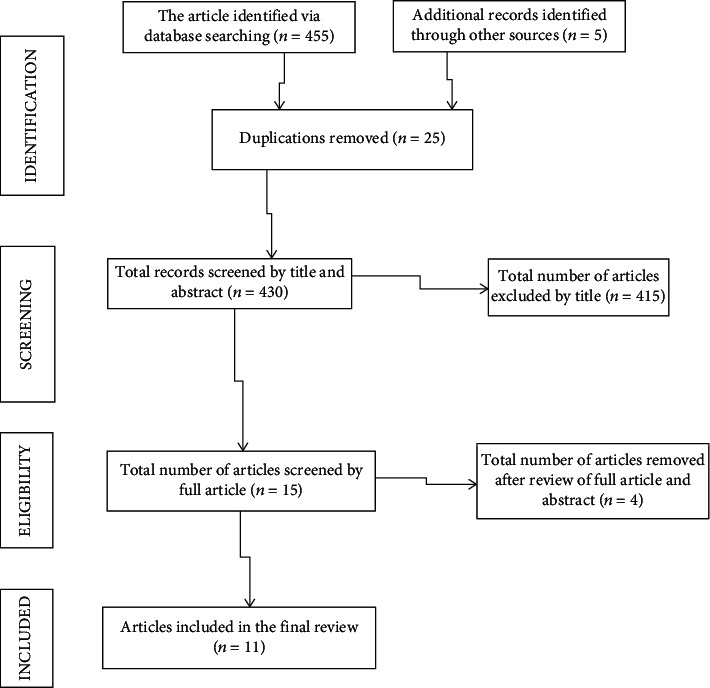
Flow diagram of study selection for systematic review and meta-analysis of depression and associated factors among adult HIV/AIDS patients in Ethiopia.

We found 455 articles through database searching (Google Scholar, African Journals Online, PubMed/MEDLINE, Hinari, Embase, Scopus, and Ethiopian university repository online). And 430 articles remained after removal of duplications. After reading of the abstract and titles, 415 articles were excluded and 4 articles were excluded after reviewing the full article and abstract. Finally, 11 articles that fulfill the inclusion criteria were used to determine pooled prevalence of depression and associated factors among adult HIV-positive patients in Ethiopia (Figure 1).

### 3.2. Characteristics of Searched Studies

A total of 11 cross-sectional studies that assessed depression prevalence and associated factors among 4642 adult HIV/AIDS patients receiving ART in Ethiopia were included in this systematic review and meta-analysis. Based on the regional distribution of searched articles, three articles were from Amhara, Oromia, and South National and Nationalities of People's Region (SNNPR) and the remaining two were from Tigray and Harar region with a total of five regions in Ethiopia included (Table 1).

### 3.3. Pooled Depression Prevalence among Adult HIV/AIDS Patients on Antiretroviral Therapy

**Figure 2:**
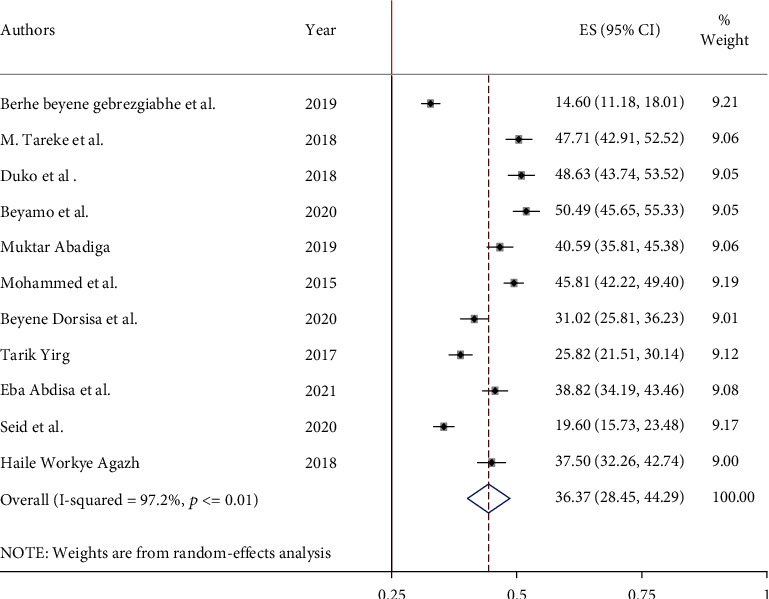
Pooled prevalence of depression among adult HIV/AIDS patients attending antiretroviral therapy.

The highest prevalence of depression among adult HIV/AIDS patients on antiretroviral therapy was 50.5% conducted in SNNPR, and the lowest was 14.6% observed in the Tigray region. The national pooled depression prevalence was 36.3% (95% CI: 28.4%, 44.2%) among adult HIV/AIDS patients receiving ART in Ethiopia (Figure 2).

### 3.4. Subgroup Analysis of Depression among Adult HIV/AIDS Patients on Antiretroviral Therapy

**Figure 3:**
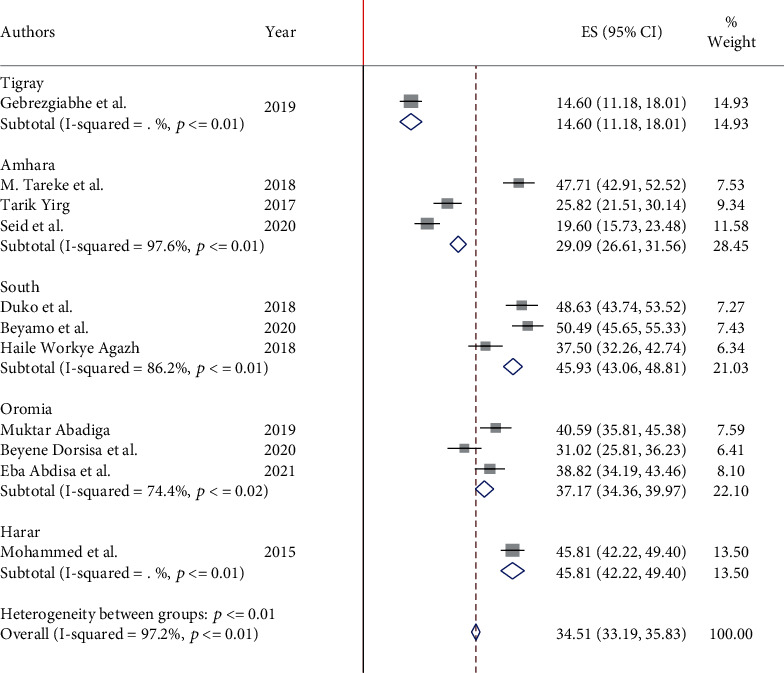
Forest plot showing the subgroup analysis of depression among adult HIV/AIDS patients attending antiretroviral therapy by region.

On subgroup analysis of depression among adult HIV/AIDS patients on antiretroviral therapy to compare the difference across regions of Ethiopia, we found the highest prevalence in Harar region which is 45.8% (95% CI: 28.5, 44.3). Next to this are South National and Nationalities of People's Region (SNNPR) 45.6% (95% CI: 37.8, 53.4), Oromia 36.9% (95% CI: 31.4, 42.5), and Amhara 31% (95% CI: 14.95, 47.04), respectively. However, the lowest prevalence was in the Tigray region (Figure 3).

### 3.5. Heterogeneity and Publication Bias

**Figure 4:**
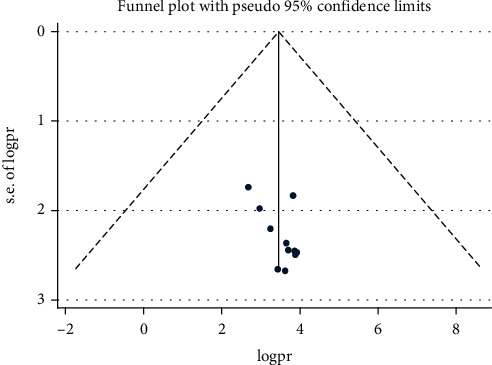
Funnel plot for the prevalence of depression among adult HIV/AIDS patients on antiretroviral therapy.

The *I*^2^ (variation in ES attributable to heterogeneity) test result revealed that there was considerable heterogeneity with *I*^2^ = 97.2, at *p* value ≤ 0.01. The funnel plot result revealed a symmetric distribution of the included studies through inspection, which implied that there was no potential publication bias (Figure 4).

### 3.6. Sensitivity Analysis

**Figure 5:**
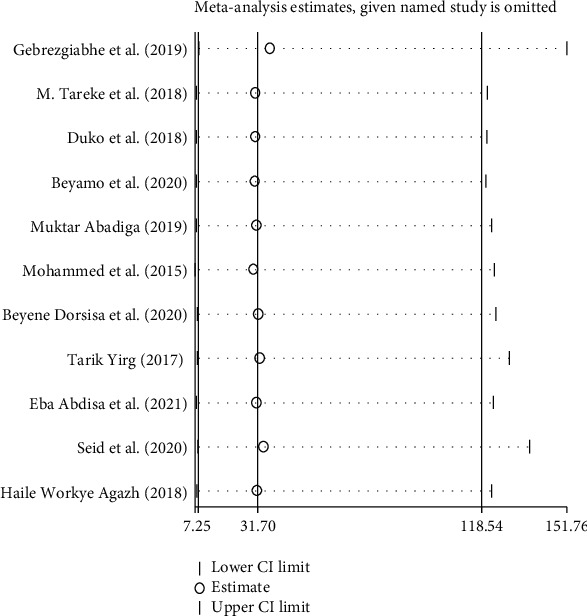
Sensitivity test of the prevalence of depression among adult HIV/AIDS patients on antiretroviral therapy in Ethiopia.

We performed the test using a random effect, and the result revealed that no single study influenced the overall pooled prevalence of depression among adult HIV/AIDS patients on antiretroviral therapy (Figure 5).

### 3.7. Factors Associated with Depression among Adult HIV/AIDS Patients

Widowed marital status, poor medication adherence, low social support, CD4 count ≤ 200, having adverse drug reactions, presence of opportunistic infections, and perceived social stigma were significantly associated with depression among adult HIV/AIDS patients at ART clinics in Ethiopia.

In this review, adult HIV/AIDS patients whose CD4 count was less than 200 were five times more likely to get depressed than those with their CD4 count greater than 200 (AOR = 5.1; 95% CI: 2.89, 8.99).

Adult HIV/AIDS patients whose marital status was widowed were four times more likely to develop depression than others (AOR = 3.7; 95% CI: 2.394, 5.789). Depression was also double on adult HIV/AIDS patients who had poor medication adherence than those with good medication adherence (AOR = 2.3; 95% CI: 1.63, 3.15). The other factor significantly associated with depression among adult HIV/AIDS patients is poor social support (AOR = 2.986; 95% CI: 2.139, 4.169). Those HIV/AIDS patients who had perceived stigma and had opportunistic infection were three times more likely to develop depression than their counterparts (AOR = 2.938; 95% CI: 2.305, 3.743; AOR = 3.01; 95% CI: 2.182, 4.151, respectively). Additionally, those adult HIV/AIDS patients who have adverse drug reactions were four times more likely to be depressed than those who did not have adverse drug reactions.

## 4. Discussion

In this systematic review and meta-analysis, the national pooled prevalence of depression among adult HIV/AIDS patients attending ART clinics of Ethiopia was 36.3% (95% CI: 28.4%, 44.2%). This finding is comparable with the systematic review conducted in east Africa (38%) [14], sub-Saharan Africa (35.8%) [20], Lahore, Pakistan (32.2%) [21], South Africa (37.6%) [15], and India (40%) [22].

However, our finding was lower than studies conducted in Uganda (47%) [23], Nigeria (56.7%) [24], Black Americans (58%) [25], Guru Teg Bahadur Hospital, Delhi [26], Sudan (63.1%) [27], and Brazil (53.5%) [28]. The difference might be because studies conducted in Nigeria, Brazil, and America are from countries that have resources and standards in their healthcare system giving depression screening and management as basic HIV care service. And in Uganda, the study participants were from advanced HIV/AIDS cases having a direct relationship with advanced stage and occurrence of depression. The other possible justification might be the sociocultural differences between study countries.

This finding was higher than that of the studies conducted in France (28.1%) [29], southwest regional hospital of Cameroon (26.7%) [30], and Malawi (18.9%) [31] and studies conducted in three countries of Africa (Kenya, Namibia, and Tanzania) [32]. This discrepancy might be due to sampling size difference, tool difference, and study design difference. A study in Cameroon used a large sample size [30]. This study is a systematic review, but studies from France, Cameroon, and three African countries were cross-sectional.

In this systematic review, adult HIV-positive patients whose CD4 count was less than or equal to 200 were five times more likely to be depressed than their counterparts. The finding was consistent with previous studies conducted in [30, 31, 33]. The possible justification for this might be like the patients' CD4 count decreased stage of HIV/AIDS becomes advanced, and they manifest more typical signs of HIV infection. As a result, they become more depressed. The present study showed that adult HIV/AIDS patients with widowed marital status were four times more depressed than others. This finding agreed with studies conducted in southern India [34]. The possible explanation could be loss of partnership and unstable marriage predisposes individuals for sadness and loneliness, which leads to depression.

In this study, depression was double on adult HIV/AIDS patients who had poor medication adherence than those with good medication adherence. This finding is consistent with previous studies [30, 32, 35, 36]. It might be that patients who had poor medication adherence leads to increased viral load and immune suppression. As a result, poor health outcomes and depression develop in those individuals. However, it is difficult to conclude which comes first, depression or poor adherence, due to the nature of the studies.

Additionally, adult HIV/AIDS patients who had poor social support were three times more likely to be depressed than their counterparts. This finding is consistent with previous studies in Canada, America, Cameroon, Delhi, North Central Nigeria, and China [26, 30, 37–40]. Social support and good community relationship provide a crucial contribution to good health, thereby reducing depression occurrence. However, social isolation leads to more stress and hurt mental health, and depression symptoms may manifest in those individuals.

The odds of depression were two times in HIV/AIDS patients who had perceived stigma than their counterparts. This finding is consistent with studies conducted in China [41], Botswana [42], systematic review in east Africa [14], and Pakistan [21]. It is due to the fact that patients who feel stigmatized by the community related to HIV/AIDS may isolate themselves from the community, and consequently, depression may develop. In addition, people with HIV/AIDS feared gossip from others, internalized stigma, which leads to depression.

This systematic review and meta-analysis showed that adult HIV/AIDS patients having opportunistic infections were three times more likely to develop depression than their counterparts. Opportunistic infection in HIV/AIDS patients exposes them to recurrent hospitalization that affects the economy and psychosocial wellbeing of the patient. In addition, the opportunistic infection had a synergistic effect on HIV/AIDS disease progression to the advanced stage. As the stage becomes advanced, patients with HIV/AIDS become depressed due to fear of death. This finding is in line with a study conducted in east Africa [14]. Adult HIV/AIDS patients who faced adverse drug reactions were four times more likely to be depressed than those who did not have adverse drug reactions. Because adverse drug side effects interfere with the optimal functions of people living with HIV/AIDS, they have fear of toxicities of the drug, become hopeless, and finally get depressed. This finding is consistent with studies conducted in China [40] and Asia [21].

## 5. Conclusions and Recommendations

More than one-third of adult HIV/AIDS patients in ART clinics developed depression in our systematic review. Depression was significantly higher among adults on ART who have widowed marital status, poor medication adherence, low social support, CD4 count ≤ 200, adverse drug reactions, presence of opportunistic infections, and perceived social stigma. Based on the finding, routine screening and integrated management of depression comorbidity with HIV-positive patients are vital to reduce its consequences. Therefore, it is better to integrate HIV care services with equitable and sustainable mental health services and give conscious attention to these groups to act on the prevention. This finding can be used as baseline data for policymakers to integrate mental health with HIV clinics. Clinicians should link cases to the mental health clinic side to reduce suicidal risks, improve ART drug adherence, and enhance the quality of life of HIV-positive patients. For future researchers, it is better to include community-based studies to get a more representative picture of the community at large.

## 6. Limitations

Since all articles in this review were cross-sectional studies, it is impossible to establish cause and effect. All studies included in this review were institutionally based; it is difficult to conclude for the community. And all regions from the country were not included; it might lack representativeness of the finding for all regions of Ethiopia. Because the number of studies included in our subgroup analysis was small, the estimate of precision might be reduced.

## Figures and Tables

**Table 1:** Description of the 11 studies included in the systematic review and meta-analysis of depression among adult HIV/AIDS patients on antiretroviral therapy.

Name of authors	Year	Region	Sample size	Cases/prevalence (%)	Design	Tools used	Response rate	Associated variables
Gebrezgiabher et al.	2019	Tigray	411	60/14.6	CS	PHQ-9	97.6%	Adverse drug reaction Social support WHO stage
Tareke et al.	2018	Amhara	415	198/47.71	CS	HAD-D	98%	Drug adherence Social support WHO stage
Duko et al.	2018	South	401	195/48.6	CS	PHQ-9	96.2%	CD4 count < 200 Social support Perceived stigma
Bayamo et al.	2020	South	410	207/50.49	CS	PHQ-9	98.3%	Drug adherence Opportunistic infection Perceived stigma
Abadiga	2019	Oromia	404	164/40.9	CS	PHQ-9	97.3%	Adverse drug reaction Opportunistic infection Social support Perceived stigma
Mohammed et al.	2015	Harar	740	339/45.81	CS	PHQ-9	97%	Widowed marital status Perceived stigma
Beyene Dorsisa et al.	2020	Oromia	303	94/31.02	CS	PHQ-9	100%	Widowed marital status Opportunistic infection
Tarik Yirg	2017	Amhara	395	102/25.82	CS	PHQ-9	98%	Drug adherence Perceived stigma WHO stage
Eba Abdisa et al.	2021	Oromia	425	165/38.82	CS	PHQ-9	90.1%	CD4 count < 200 Social support Perceived stigma
Seid et al.	2020	Amhara	403	79/37.5	CS	PHQ-9	93.5%	CD4 count < 200 Widowed marital status Drug adherence Opportunistic infections
Haile Workye Agazh	2018	South	328	123/36.37	CS	PHQ-9	96.5%	Social support Perceived stigma

## Data Availability

Full datasets and other materials about this study could be obtained from the corresponding author upon reasonable request.

## Abbreviations

AIDS: Acquired immunodeficiency disease syndrome
ART: Antiretroviral treatment
AOR: Adjusted odds ratio
CD4: Cluster of cell differentiation 4
CI: Confidence interval
CS: Cross-sectional
HIV: Human immunodeficiency virus.
HIV/AIDS: Human immunodeficiency virus/acquired immunodeficiency disease syndrome
SNNPR: South National and Nationalities of People's Region
PHQ-9: Patient health questionnaire.
